# Self-report health-related quality of life among children and adolescents from Bogotá, Colombia. The FUPRECOL study

**Published:** 2017-03-30

**Authors:** Darío Fernando Gaitán-López, Jorge Enrique Correa-Bautista, Stefano Vinaccia, Robinson Ramírez-Vélez

**Affiliations:** 1 Centro de Estudios en Medición de la Actividad Física (CEMA), Escuela de Medicina y Ciencias de la Salud, Universidad del Rosario, Bogotá D.C, Colombia.; 2 Universidad del Sinu, Facultad de Ciencias de la Salud. Programa de Psicología. Montería, Colombia

**Keywords:** Personal satisfaction, surveys and questionnaires, quality of life, child, adolescents, psychometrics, health status, mental health

## Abstract

**Objective:**

To describe by self-report the HRQoL among schoolchildren from Bogotá, Colombia belonging to the FUPRECOL study.

**Methods:**

A cross-sectional study in 3,245 children and 3,354 adolescents, between 9 and 17.9 years old, participated in the study. Spanish version of the EQ-5D-Y was self-assessment. Percentages of missing values and reported problems were calculated. The data was analyzed by measurement of central tendency stratified by age group, and to compare them to international references.

**Results:**

A total of 58.3%, (n= 3,848) were women. In all ages, the HRQoL was higher in boys than in girls. To compare by sex, the dimensions of the EQ-5D-Y "feeling worried, sad or unhappy" and "having pain or discomfort", showed the highest frequency among women. Overall, our HRQoL were higher than South Africa, Germany and Italy references.

**Conclusion:**

The HRQoL was higher in boys than in girls The HRQoL. The dimensions of the EQ-5D-Y "feeling worried, sad or unhappy" and "having pain or discomfort", showed the highest frequency. The HRQoL by age and sex may be used in the evaluation of the health perceived among schoolchildren from Bogotá.

## Resumen

**Objetivo:**

Describir por autoreporte la calidad de vida relacionada con la salud (CVRS) en una población escolar de Bogotá, Colombia, pertenecientes al estudio FUPRECOL.

**Métodos:**

Estudio descriptivo y transversal, en 3,245 niños y 3,354 adolescentes, entre 9 y 17.9 años de edad, de 24 instituciones educativas oficiales de Bogotá, Colombia. Se aplicó de manera auto-administrada del instrumento de CVRS infantil EQ-5D-Y proxy, versión validada al castellano. Se analizaron los datos por medidas de tendencia central y se realizó una comparación de los observados en Colombia con estudios internacionales.

**Resultados:**

De la población evaluada, el 58.3% (n= 3,848), fueron mujeres. En general, se observaron puntuaciones elevadas en la CVRS en niños y adolescentes de ambos sexos. Al comparar por género, las dimensiones del EQ-5D-Y proxy "sentirse triste/preocupado o infeliz" y "tener dolor/malestar", presentaron la mayor frecuencia de respuesta en el grupo de las mujeres. Al comparar los resultados de este estudio, por grupos de edad, con trabajos internacionales de niños y adolescentes, se observó que las puntuaciones del EQ-5D-Y proxy fueron superiores a los reportados en Suráfrica, Alemania e Italia.

**Conclusión:**

Los niños acusan menor porcentaje de problemas en todas las dimensiones que los adolescentes. Las dimensiones relativas a "dolor/malestar" y "sentirse triste/preocupado o infeliz", fueron en las que se indicaron más problemas. Se presentan valores de la CVRS según edad y sexo que podrán ser usados en la evaluación de la salud percibida en el ámbito escolar en Bogotá, Colombia.

## Introduction

In recent years, the term health-related quality of life (HRQL) is being positioned as an indicator of physical and mental wellbeing since childhood and adolescence ^1^. This concept emerges as a useful indicator of the aggregate state of health, which bears in mind the individual's self-perception as a requirement in the health evaluation ^2^. Currently, the HRQL is defined as the *"subjective perception, influenced by the current state of health, of the capacity to perform activities deemed important for the individual"* ^3^ and authors, like Ramírez-Vélez *et al*. ^4^, propose that monitoring of HRQL in different populations will permit population identification of altered physical and/or mental health as an input to guide policies or interventions to improve their collective health.

To estimate this important indicator of the state of health, generic and specific instruments have been developed to measure child and adolescent HRQL starting at 8 years of age through self-report questionnaires ^4^. Among the most used, the Health Related Quality of Life Questionnaire for Children and Young People and their Parents (KIDSCREEN-27) ^5^, the Child Health and Illness Profile (CHQ) ^6^, the *Autoquestionnaire Qualité de Vie-Enfant-Imagé* (AUQUEI) ^7^, the Pediatric Quality of Life Inventory (PedsQL) ^8^, and the EQ-5D-Y ^9^ ^,^ ^10^; all used and validated in Hispanic pediatric population. The EQ-5D-Y proxy is a generic and practical questionnaire, of easy comprehension and application, which provides values in various dimensions of self-perceived health, as well as a visual scale that represents the current state of health ^9^ ^,^ ^10^. The EQ-5D-Y proxy questionnaire, originally developed by Wille *et al*. ^9^, was adapted into Spanish by Olivares *et al*. ^11^, and recently a "*proxy*" version was published by Gusi *et al.* ^10^, on Spanish pediatric and young population.

Previously, substantial differences have been shown in the self-perception of HRQL in Hispanic, European, and American children and adolescents, thus, suggesting the need to have ethnic-specific values that describe and characterize this important health indicator ^5^ ^-^ ^10^. It has also been described that the young Latin American population has particular characteristics in its growth, development, and physical health, a product of the inbreeding of European, Amerindian, and African ancestors, making it difficult to establish a clear differentiation between the influence of the environmental and genetic factors and the self-perception of health ^12^ ^,^ ^13^. 

In spite of increased studies on HRQL, few investigations are aimed at the Latin population, specifically within the epidemiological and educational context of Colombia ^14^. Thereby, the objective of this study was to describe the perceived quality of life in children and adolescents with the Spanish version of the EQ-5D-Y proxy, to later use it in the Colombian school context. A second objective was the comparison between the EQ-5D-Y proxy values observed in this study and international reports.

## Materials and Methods

### Study type and population

We performed cross-sectional analyses of baseline data from participants in The FUPRECOL study (In Spanish *Asociación de la Fuerza Prensil con Manifestaciones Tempranas de Riesgo Cardiovascular en Niños y Adolescentes Colombianos*), which focused on the associations between fitness, health and non-communicable diseases. We have recently published a complete description of The FUPRECOL Study design, methods, and primary outcomes for our current cohort, residing in the metropolitan area of the District of Bogotá, Colombia (2,480 masl). The population reference used 546,000 registration records for 2013, provided by the District's Secretary of Education. This calculation required using the equation for finite samples:



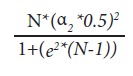



Where, N = 546,000; (*e*) Precision= 1.5%; α = 0.05, 95% confidence interval. The sample size calculated was of 4,235. The sampling was conducted through convenience in order of arrival to the data collection point. To diminish bias because it was a non-probabilistic sample, sampling weight *- a posteriori -* was assigned to each participant, calculated from the stratification by age groups (± 1 year). This kept in mind that "N" is the population size and "n" is the sample size, whose inclusion probabilities were πi = n/N and the weighted samplings ωi = n/N. The study excluded students with clinical diagnosis of physical, sensory, and intellectual disability, non-communicable diseases, like type 1 or 2 diabetes, cardiovascular disease, autoimmune disease, cancer, pregnancy, alcohol or drug abuse, and - in general - pathologies not directly related to nutrition. Effective exclusion was carried out *a posteriori*, without the participants' knowledge; therein, respecting their dignity. 

### Procedures

Prior to the study's measurements, the researchers and the physical education teachers held 10 theoretical-practical sessions to standardize the evaluation process. Then, the project was introduced to the educational community in the institutions selected: rectors, faculty, parents, and students through an informative letter, which explained the nature and objectives of the research. Thereafter, informed signed consent and written acceptance were obtained.

### Measurement of childhood HRQL (EQ-5D-Y Proxy)

This work applied the EQ-5D-Y proxy version published in Spanish used with a Spanish pediatric and youth population by Gusi *et al*. ^10^, (open version http://www.euroqol.org/about-eq-5d.html). This simple, short version of easy administration provides results from five health dimensions, as well as a single or index value, which can be used to assess the self-perceived state of health ^10^. According to Gusi *et al*. ^10^, the EQ-5D-Y proxy includes five items that inquire on mobility, self-care, performance of habitual activities, the presence of pain or discomfort, and feeling sad, worried or unhappy. Each question includes three response levels in function of the difficulty or problem in each dimension (without problems, some problems, or many problems). In addition, the EQ-5D-Y also includes a visual analogue scale (VAS) in which the subject (male or female) must perform a global evaluation of his/her state of health in a scale from 0 to 100, where 0 represents the worst state of health imagined, and 100 the best state of health imagined ^10^. This study applied the EQ-5D-Y proxy version in paper and self-administered. The time for the self-evaluation was on average 6.0 ± 2.1 min.

### Ethical considerations

The study protocol was explained verbally to the participants and their parents/guardians before they gave their written consent. Participation in the study was fully voluntary and anonymous, with no incentives provided to participants. The Review Committee for Research on Human Subjects at the University of Rosario (code No. CEI-ABN026-000262) approved all study procedures. The protocol was in accordance with the latest revision of the Declaration of Helsinki and current Colombian laws governing clinical research on human subjects (Resolution 008430/1993 Ministry of Health).

### Statistical analysis

Information processing and analysis was performed in the Statistical Package for Social Science*^(r)^* software, version 22 (SPSS; Chicago, IL, USA). Normality tests were run through Kolmogorov-Smirnov tests. Continuous values were expressed as tendency and dispersion measures and proportions were expressed in percentages. Chi square (X^2)^ tests were applied for differences between proportions by gender, age, and age groups (children vs. adolescents). A Cronbach's α index was obtained for internal consistency terms for each of the five aspects of the EQ-5D-Y proxy in relation to its total. All the analyses were adjusted by the sampling weights, bearing in mind the sample design and the population expansion factors. 

## Results

Of the 6,950 individuals asked to participate in the study, 6,599 were of school age: 3,245 males (9 and 12 years of age) and 3,354 were adolescents (13 and 17 years of age), who filled out the EQ-5D-Y proxy survey (94.9% rate of participation). Of the population surveyed, 41.7% (n= 2,751) were males and 58.3% (n= 3,848) women (mean age 12.7 ± 2.3 years) (Table 1). 

**Table 1:** Characteristics among a sample of children and adolescents by sex and age from Bogota, Colombia (n= 6,599)

Age	Boys		Girls		Total	
	n	%	n	%	n	%
9+	185	41.6	260	58.4	445	6.7^a^
10+	410	39.1	638	60.9	1.048	15.9^b^
11+	386	39.0	605	61.0	991	15.0^c^
12+	323	42.4	438	57.6	761	11.5^d^
13+	319	44.4	399	55.6	718	10.9^e^
14+	305	38.5	488	61.5	793	12.0^f^
15+	328	43.4	427	56.6	755	11.4^g^
16+	286	44.5	356	55.5	642	9.7^h^
17+	209	46.9	237	53.1	446	6.8^i^
All of them	2,751	41.7	3,848	58.3	6,599	100^j^

Date missing: a = 43, b= 68, c = 58, d = 39, e = 41, f = 43, g = 33, h = 24, i = 10, j = 359

Table 2 presents the frequency of the problems indicated by Colombian males and adolescents, according to age and gender. In general, males show better quality of life than women in the majority of dimensions, especially in the dimensions "pain/discomfort" and "feeling sad/worried or unhappy" (*p*= 0.45).

**Table 2:** Percentage of problems reported in the EQ-5D-Y by sex and age

Dimensions	Boys									Girls								
	9+	10+	11+	12+	13+	14+	15+	16+	17+	9+	10+	11+	12+	13+	14+	15+	16+	17+
Mobility																		
No problems	174 (94.1)	387 (94.4)	371 (95.9)	304 (94.1)	304 (95.3)	290 (95.1)	290 (95.1)	269 (94.1)	196 (93.3)	245 (94.2)	593 (92.8)	577 (95.4)	406 (92.7)	364 (90.8)	448 (91.1)	448 (91.1)	323 (90.7)	214 (90.3)
Some problems	8 (4.3)	21 (5.1)	11 (2.8)	15 (4.6)	14 (4.4)	14 (4.6)	14 (4.6)	17 (5.9)	12 (5.7)	15 (5.8)	44 (6.9)	28 (4.6)	30 (6.8)	31 (7.7)	35 (7.1)	35 (7.1)	30 (8.4)	23 (9.7)
A lot of problems	3 (1.6)	2 (0.5)	5 (1.3)	4 (1.2)	1 (0.3)	1 (0.3)	1 (0.3)	0 (0.0)	2 (1.0)	0 (0.0)	2 (0.4)	0 (0.0)	2 (0.5)	6 (1.5)	9 (1.8)	9 (1.8)	3 (0.8)	0 (0.0)
Self-care																		
No problems	180 (96.8)	403 (98.3)	382 (98.5)	318 (98.8)	316 (98.4)	298 (98.3)	298 (98.3)	284 (99.3)	205 (97.2)	254 (96.6)	622 (97.3)	600 (98.7)	430 (97.1)	393 (98.3)	478 (97.2)	478 (97.2)	354 (98.9)	237 (100)
Some problems	6 (3.2)	2 (0.5)	4 (1.0)	3 (0.9)	3 (0.9)	3 (1.0)	3 (1.0)	1 (0.3)	6 (2.9)	8 (3.0)	15 (2.3)	3 (0.5)	8 (1.8)	3 (0.8)	6 (1.2)	6 (1.2)	4 (1.1)	0 (0.0)
A lot of problems	0 (0.0)	5 (1.2)	2 (0.5)	1 (0.3)	2 (0.6)	2 (0.7)	2 (0.7)	1 (0.3)	0 (0.0)	1 (0.4)	2 (0.3)	5 (0.8)	5 (1.1)	4 (1.1)	8 (1.8)	8 (1.6)	0 (0.0)	0 (0.0)
Doing usual activities																		
No problems	176 (94.6)	383 (93.6)	373 (95.6)	306 (95.3)	312 (96.9)	289 (94.4)	289 (94.4)	275 (96.2)	199 (94.3)	252 (96.2)	601 (94.3)	582 (95.6)	417 (94.3)	376 (93.5)	455 (92.1)	455 (92.1)	324 (90.8)	215 (90.7)
Some problems	8 (4.3)	19 (4.6)	16 (4.1)	14 (4.4)	8 (2.5)	15 (4.9)	15 (4.9)	11 (3.8)	10 (4.7)	8 (3.1)	29 (4.6)	24 (3.9)	23 (5.2)	23 (5.7)	35 (7.1)	35 (7.1)	32 (9.0)	21 (8.9)
A lot of problems	2 (1.1)	7 (1.7)	1 (0.3)	1 (0.3)	2 (0.6)	2 (0.7)	2 (0.7)	0 (0.0)	2 (1.0)	2 (0.8)	7 (1.1)	3 (0.5)	2 (0.4)	3 (0.7)	4 (0.8)	4 (0.8)	1 (0.3)	1 (0.4)
Having pain or discomfort																		
No problems	164 (88.2)	347 (84.6)	338 (86.7)	270 (83.6)	273 (84.8)	257 (84.5)	257 (84.5)	223 (78.2)	182 (86.3)	215 (81.7)	515 (80.7)	458 (75.7)^a^	345 (78.1)^a^	277 (68.7)^a^	338 (68.3)^a^	338 (68.3)^a^	224 (62.7)^a^	137 (57.8)^a^
Some problems	19 (10.2)	56 (13.7)	47 (12.1)	49 (15.2)	47 (14.6)	45 (14.8)	45 (14.8)	61 (21.4)	26 (12.3)	44 (16.7)^a^	112 (17.6)^a^	140 (23.1)^a^	90 (20.4)^a^	119 (29.5)^a^	145 (29.3)^a^	145 (29.3)^a^	127 (35.6)^a^	95 (40.1)^a^
A lot of problems	2 (1.1)	7 (1.7)	5 (1.3)	4 (1.4)	2 (0.6)	2 (0.7)	2 (0.7)	1 (0.4)	3 (1.4)	4 (1.5)	11 (1.7)	7 (1.2)	7 (1.6)	7 (1.7)	12 (2.4)	12 (2.4)	6 (1.7)	5 (2.1)
Feeling worried, sad, or unhappy																		
No problems	162 (87.1)	354 (87.0)	322 (83.0)	259 (80.4)	249 (77.3)	229 (75.1)	229 (75.1)	211 (73.8)	158 (74.9)	224 (85.2)	529 (82.9)	467 (77.1)^a^	328 (73.9)^a^	261 (64.9)^a^	314 (63.4)^a^	314 (63.4)^a^	197 (55.0)^a^	133 (56.1)^a^
Some problems	19 (10.2)	46 (11.3)	59 (15.2)	54 (16.8)	69 (21.4)	68 (22.3)	68 (22.3)	66 (23.1)	49 (23.2)	32 (12.2)	96 (15.0)^a^	124 (20.5)^a^	104 (23.4)^a^	128 (31.8)^a^	158 (31.9)^a^	158 (31.9)^a^	140 (39.1)^a^	99 (41.8)^a^
A lot of problems	5 (2.7)	7 (1.7	7 (1.8	9 (2.8	4 (1.2)	8 (2.6)	8 (2.6)	9 (3.1)	4 (1.9)	7 (2.7)	13 (2.0)	15 (2.5)	12 (2.7)	13 (3.2)	23 (4.6)	23 (4.6)	21 (5.9)	5 (2.1)

^a^ Significant between-sex differences for χ2* (p <0.05)

Tables 3 and ^4^ show the frequency of the problems indicated during childhood and adolescence in males and females, respectively. In general, children (9 to 12 years of age) have a lower percentage of problems in all the dimensions than the adolescents (13 to 17 years of age). Dimensions related to "pain/discomfort" and "feeling sad/worried or unhappy" indicated more problems, especially in the group of adolescent females (*p* <0.41).

**Table 3:** Percentage of problems reported in the EQ-5D-Y in boys

Dimensions	Children 9-12 years		Adolescents 13-17 years		Total	
	n	%	n	%	n	%
Mobility						
No problems	1,236	94.7	1,373	94.8	2,609	94.8
Some problems	55	4.2	70	4.8	125	4.5
A lot of problems	13	0.9	4	0.2	17	0.6
Self-care						
No problems	1,283	98.2	1,432	98.8	2,715	98.5
Some problems	15	1.1	12	0.8	27	0.9
A lot of problems	8	0.6	4	0.2	12	0.4
Doing usual activities						
No problems	1,238	95.0	1,395	95.8	2,633	95.0
Some problems	57	4.3	55	3.7	112	4.0
A lot of problems	8	0.6	5	0.3	13	0.4
Having pain or discomfort						
No problems	1,119	85.4	1,210	83.0^a^	2,329	84.2
Some problems	171	13.0	237	16.2^a^	408	14.7
A lot of problems	19	1.4	10	0.6	29	1.0
Feeling worried, sad, or unhappy						
No problems	1,097	84.1	1,095	75.0^a^	2,192	79.3
Some problems	178	13.6	334	22.8^a^	512	18.5
A lot of problems	28	2.1	30	2.0	58	2.0

^a^ Significant between-sex differences for χ2* (p<0.05)

**Table 4:** Percentage of problems reported in the EQ-5D-Y in girls

Dimensions	Children 9-12 years		Adolescents 13-17 years		Total	
	n	%	n	%	n	%
Mobility						
No problems	1,821	93.8	1,736	91.0	3,557	92.4
Some problems	117	6	158	8.2	275	7.1
A lot of problems	3	0.1	13	0.6	16	0.4
Self-care						
No problems	1,906	97.6	1,885	98.7	3,791	98.1
Some problems	34	1.7	15	0.7	49	1.2
A lot of problems	12	0.6	9	0.4	21	0.5
Doing usual activities						
No problems	1,852	95.1	1,766	92.2	3,618	93.7
Some problems	84	4.3	142	7.4	226	5.8
A lot of problems	10	0.5	7	0.3	17	0.4
Having pain or discomfort						
No problems	1,533	78.6	1,237	64.3^a^	2,770	71.5
Some problems	386	19.8	645	33.5^a^	1.031	26.6
A lot of problems	29	1.4	39	2.0	68	1.7
Feeling worried, sad, or unhappy						
No problems	1,548	79.3	1,136	59.2^a^	2,684	69.3
Some problems	356	18.2	699	36.4^a^	1,055	27.2
A lot of problems	47	2.4	83	4.3^a^	130	3.3

^a^ Significant between-sex differences for χ2* (p<0.05)

The results of the global evaluation of the state of health indicated in the VAS in Colombian children and adolescents are shown in Table 5. Both in males and females, higher values of self-perception of health were observed in the group of children 9-12 years of age (median 95, CI range 85-100) against the group of adolescents (median 90, CI range 80-99).

**Table 5:** Overall assessment of the health status by Visual Analog Scale in Colombian children and adolescents

Group	Children 9-12 years		Adolescents 13-17 years		Total	
	Median	Range	Median	Range	Median	Range
Boys	95	85-100	90	80-99	95	85-100
Girls	95	90-100	90	80-95	90	80-100

The EQ-5D-Y and the proxy version also include a visual analogue scale (VAS). A scale from 0 to 100, with 0 representing the worst and 100 the best health state he or she can imagine*.*

Table 6 compares the results from this study (dimensions and VAS) of the EQ-5D-Y proxy version with data from other international studies from Spain ^10^ ^,^ ^15^ ^,^ ^16^, Australia ^17^, Germany ^16^, Italy ^16^, South Africa ^16^, and Sweden ^16^. In the "mobility" dimension, the scores for this work were above those reported in Germany ^16^ and South Africa ^16^. The "self-care" dimension showed higher values than the works from Spain ^10^ ^,^ ^15^ ^,^ ^16^, Italy ^16^, and South Africa ^16^. The "habitual activities" dimension had similar behavior when compared to school-age children from Spain ^10^ ^,^ ^15^ ^,^ ^16^, Germany ^16^, Italy ^16^, and South Africa ^16^. Similarly, the dimensions of "pain/discomfort" and "feeling sad, worried or unhappy" showed values above those indicated by children and adolescents from Germany ^16^, Italy ^16^, South Africa ^16^, and Sweden ^16^. 

**Table 6:** Comparison of problems reported in the EQ-5D-Y among a sample of children and adolescents from cited studies*

Characteristics/Dimensions	FUPRECOL study	Australia^18^	Spain (Barcelona) ^15^	Spain (Extremadure) ^16^	Spain (Extremadure) ^10^	Germany^16^	Italia^16^	SouthAfrica^16^	Sweden^16^
Year of publication	2015	2015	2010	2015	2014	2010	2010	2010	2010
Sample, (n)	6,599	2,020	973	923	620	756	415	258	407
Age of participation, (years)	9-17	11-17	8-18	8-18	6-17	10-18	8-15	8-18	8-16
Method of administration	Paper	On-line	Paper	Paper	Paper	Paper	Paper	Paper	Paper
Mobility									
No problems	93.2	89.6	95.7	93.3	96.8	92.7	93.7	89.4	97.6
Some problems	6.0	9.5	4.3	6.3	2.6	7.3	6.0	10.1	2.4
A lot of problems	0.7	0.7	0.0	0.4	0.6	0.0	0.3	0.5	0.0
Self-care								
No problems	98.2	95.6	98.9	96.5	96.6	98.1	95.5	96.3	99.2
Some problems	1.1	3.9	0.9	2.7	3.1	1.7	4.2	3.2	0.8
A lot of problems	0.6	0.4	0.2	0.8	0.3	0.2	0.3	0.5	0.0
Doing usual activities									
No problems	94.2	86.3	94.4	93.4	92.7	93.8	84.6	87.8	97.1
Some problems	5.1	12.5	5.0	5.9	6.1	5.9	14.6	12.2	2.9
A lot of problems	0.7	1.0	0.6	0.7	1.0	0.3	0.8	0.0	0.0
Having pain or discomfort									
No problems	76.9	62.6	80.0	80.9	84.2	66.0	61.5	58.5	75.4
Some problems	21.7	35.0	19.0	18.1	15.0	32.9	37.7	41.0	24.1
A lot of problems	1.4	2.3	1.0	1.0	0.8	1.1	0.8	0.5	0.5
Feeling worried, sad, or unhappy									
No problems	73.5	54.2	77.8	80.2	79.8	60.8	62.0	63.8	82.3
Some problems	23.6	42.7	21.4	18.0	18.9	35.4	34.1	34.0	17.2
A lot of problems	2.9	3.0	0.9	1.8	1.1	3.8	3.9	2.1	0.5
Visual analogue scale (VAS), (%)									
Median (standard deviation)	88.3 (14.0)	-	-	85.6 (16.2)	-	-	-	-	-

Lastly, the reliability analysis shows internal consistency results (Cronbach's α of 0.61) for the "mobility" dimension; (0.66) in "self-care"; (0.62) for "performing habitual activities"; (0.43) in "pain/discomfort"; and (0.69) in the dimension of "feeling sad/worried or unhappy". The Cronbach's α for the total EQ-5D-Y proxy questionnaire was found at 0.64.

## Discussion

The study had the participation of 6,599 children and adolescents of both genders, attending basic primary and basic secondary education in 24 public institutions belonging to several localities of Bogotá, Colombia. To our knowledge, this is the first report publishing the use of the EQ-5D-Y proxy in students in Colombia. Regarding the HRQL, males report better quality of life than females in most categories - especially in the dimensions of "pain/discomfort" and "feeling sad/worried or unhappy". These results are similar to those obtained by Quiceno and Vinaccia ^18^ in 686 children and adolescents from Bogotá Colombia with the "KIDSCREEN-52" childhood HRQL instrument. These authors state that males perceive better levels of HRQL than females. 

Most of the HRQL values within the school context that applied the EQ-5D-Y proxy version have shown results similar to those in this study ^15^ ^-^ ^17^. The findings in this study agree with the work published for Spanish children and adolescents ^10^ ^,^ ^15^ ^,^ ^16^, as well as with that found in other research of the same type ^17^ ^,^ ^18^. Low prevalence of serious problems was also found reported in the different dimensions of the EQ-5D-Y, results that coincide in infant/child population from European countries ^10^ ^,^ ^15^ ^-^ ^17^, South Africa ^16^, or Australia ^18^. 

With respect to age, greater frequency was found in the dimensions of "pain/discomfort" and "feeling sad/worried or unhappy", with the EQ-5D-Y proxy questionnaire, consistently and in both genders as age increased. These findings agree with that described by Gusi *et al*. ^10^, in the study to validate the EQ-5D-Y in Spanish school-age children. It has been reported that children (9-12 years of age) have a better perception of their HRQL compared to adolescents (13-17 years of age), particularly in the dimensions referring to their perception on the school environment, as well as their physical and emotional wellbeing ^19^. For Guedes *et al*. ^20^, the differences in health perception between children and adolescents may be due in part to the start of menarche and of the expected hormonal imbalance occurring in the female organism at these ages, reducing the opportunities women have to confront satisfactorily stressful events operating during this period of their lives. Although this difference of perception in children could be explained partially by the adolescent behavior, we could also inquire on the school's role and its specific contribution in this regard, as suggested by Quiceno and Vinaccia ^21^. Newman *et al*. ^22^, indicate that a lower health perception in adolescents can also be explained by "*bullying", - a very frequent situation during the second half of adolescence-,* an aspect that is quite related to the EQ-5D-Y proxy dimensions of "pain/discomfort" and "feeling sad/worried or unhappy". 

In addition, the global assessment of the state of health indicated in the VAS of the study participants showed high values of self-perception of health, especially in the group of children from 9 to 12 years of age compared to the group of adolescents from 13 to 17 years of age, in both genders. In this sense, Hernan-Gómez *et al*. ^23^, propose that during adolescence there may be a decrease of positive emotions and of vital satisfaction with a gradual increase of negative emotions that could affect the self-perception of health. This finding coincides with that reported by Urzúa ^24^, who stated that female adolescents have the worst perception with respect to their HRQL, with the physical wellbeing and, particularly, their perception of themselves being the two dimensions with less favorable evaluation. This perception of adolescent women of themselves constitutes a warning signal for health authorities, given that it could be indicating the lack of satisfaction of adolescent women with their own bodies and, thus, a higher risk for the onset of eating pathologies ^25^. In synthesis, the lower perception of HRQL during the adolescent stage could certainly be related to the complexity that characterizes this stage of development, especially in women from Bogotá, Colombia.

Regarding the administration of the EQ-5D-Y proxy instrument, "in paper and self-administered", it is practical and of easy application, suggesting the implementation of further studies in other Colombian regions and latitudes to analyze possible disparities with our results. Several authors have described life factors that are considered important for the HRQL, like family relationships and social support, general health, the functional state, and economic availability ^9^ ^,^ ^10^ ^,^ ^23^ ^-^ ^25^. 

Hence, we can state that, although new studies are needed to advance in establishing the instrument's transcultural equivalence, the results obtained constitute an important starting point to have an instrument to measure childhood HRQL useful for pediatrics and Colombian public health. The high ceiling effect (values close to 100%) of the participants may perhaps be explained in part because most of the children and adolescents were healthy and had no important health problems. For this purpose, it will be necessary to analyze the psychometric properties of the EQ-5D-Y proxy version in Colombian population.

The principal limitations of this study are those inherent to its transversal nature and type of sampling. For example, it is important to indicate that information equivalent to the components of HRQL was obtained via self-report. The self-report is a common procedure in studies with these characteristics, being the most feasible way to obtain data related to HRQL in broad populations ^2^ ^-^ ^9^ ^,^ ^23^ ^-^ ^25^. Also, the transversal approach of the data could have limited identification of differences, without being able to formulate the likelihood of the existence of inverse causality. Also, socioeconomic level was not studied, although it has been described that this variable does not seem to modify substantially the perception on HRQL in children and adolescents from both genders, except for the dimension specifically related to the perception of the family's financial resources ^25^. Among the strengths found is that the study included a large population sample and adjusted by population expansion factors of both genders, offering new perspectives on the self-perceived state of health of school-age children in Bogotá, Colombia, which should be kept in mind by those involved in the settings of planning, decision, and execution of health policies. 

## Conclusion

Favorable perception of HRQL is noted in the school-aged children evaluated. We expect that subsequent studies can establish interpretation standards in the general population and in different clinical populations, besides the study of their psychometric properties. In spite of its importance, methodological simplicity, and clinical use, the determination of HRQL is still not part of the protocols to evaluate the state of health of school-age children in Bogotá, Colombia. 
